# Application of a conversion electrode based on decomposition derivatives of Ag_4_[Fe(CN)_6_] for aqueous electrolyte batteries†

**DOI:** 10.1039/d2ra00617k

**Published:** 2022-03-29

**Authors:** Fyodor Malchik, Kaiyrgali Maldybayev, Tatyana Kan, Saule Kokhmetova, Andrey Kurbatov, Alina Galeyeva, Nufar Tubul, Netanel Shpigel, Thierry Djenizian

**Affiliations:** Al-Farabi Kazakh National University, Center of Physical–Chemical Methods of Research and Analysis Almaty 050012 Kazakhstan Frodo-007@mail.ru; Department of Chemistry and BINA – BIU Center for Nanotechnology and Advanced Materials, Bar-Ilan University Ramat-Gan 5290002 Israel; Institute of Chemistry, The Hebrew University of Jerusalem Jerusalem 9190401 Israel; Mines Saint-Etienne, Center of Microelectronics in Provence, Flexible Electronics Department 13541 Gardanne France

## Abstract

The lack of stable electrode materials for water-based electrolytes due to the intercalation and conversion reaction mechanisms encourage scientists to design new or renovate existing materials with better cyclability, capacity, and cost-effectiveness. Ag_4_[Fe(CN)_6_] is a material belonging to the Prussian blue family that can be used, as its other family members, as an electrode material with the intercalation/deintercalation reaction or conversion-type mechanism through Ag oxidation/reduction. However, due to the instability of this material in its dry state, it decomposes to AgCN and a Prussian blue residual complex. A possible reason for Ag_4_[Fe(CN)_6_] decomposition is discussed. Nevertheless, it is shown that the decomposition products of Ag_4_[Fe(CN)_6_] have electrochemical activity due to the reversible oxidation/reduction of Ag atoms in water-based electrolytes.

## Abstract 2

The lack of stable electrode materials for water-based electrolytes based on intercalation and conversion reaction mechanisms encourage scientists to design new or renovate existing materials with better cyclability, capacity, and cost-effectiveness.

## Graphical abstract

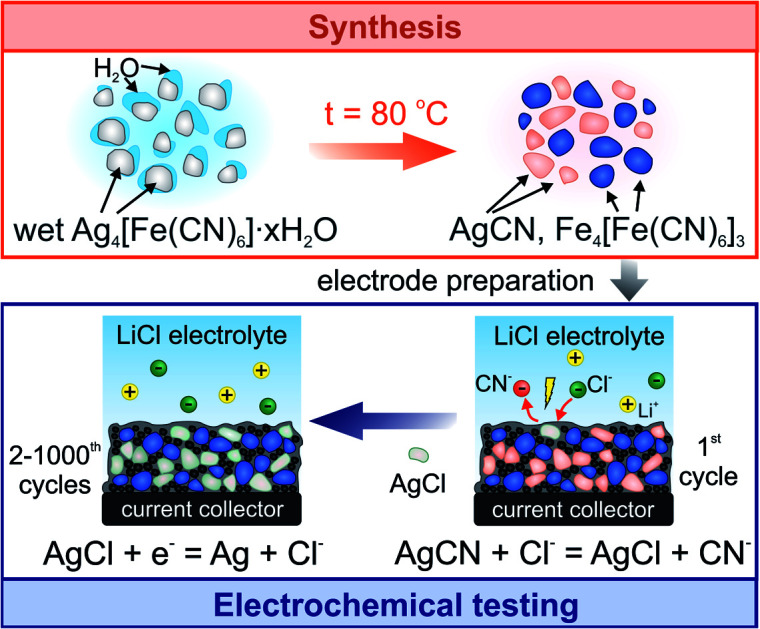

## 1. Introduction

The high global demand for rechargeable batteries actively encourages researchers to look for new cathode and anode materials to increase their performance in terms of power, energy density, stability, charge/discharge rate, *etc.*, as well as to reduce their cost. Currently, promising results have been obtained with new materials for battery electrodes based on inexpensive derivatives of the Prussian blue family of materials, with the general formula A_*x*_P[R(CN)_6_]·*x*H_2_O, where A is an alkaline cation, P is an N-coordinated transition metal cation, R is a C-coordinated transition metal cation and [R(CN)_6_] is a hexacyanometallate anion.^1–3^ Almost all representatives of the Prussian blue family are relatively inexpensive (considering the price of the precursors consisting of the common elements C, N, Fe and other transition metals) and their synthetic methods are simple, such as precipitation from aqueous solutions, and require no special heat treatment.^4^

Previous works have mostly been devoted to the electrochemistry of hexacyanoferrates, where a C-coordinated Fe reversibly shows an oxidation state varying between III and II in the potential range of 0.2–1.2 V *vs.* SHE (3.25–4.25 V *vs.* Li/Li^+^). The exact value of the working potential depends on the inductive effect of the N-coordinated transition metal ion, which make Prussian blue analogues (PBAs) ideal positive electrode materials for organic and water-based electrolyte metal-ion batteries. To compensate for the charge changes that occur during iron oxidation/reduction, a simultaneous intercalation/deintercalation of positive ions (Li^+^, Na^+^, K^+^, Ca^2+^, Al^3+^, *etc.*) occurs in the crystal lattice.^5^ There is also a wide scope of other PBAs with different C-coordinated transition metals (not only Fe) with broader values of standard potentials, which can be used as anodes for organic and water-based electrolyte batteries that present inherent reversible intercalation/deintercalation reactions of certain positive ions.^2^

In addition to the intercalation/deintercalation charge storage mechanism, there is a conversion reaction type for certain representatives of PBAs that are only used as anode materials and solely in organic electrolytes.^6^ The reason for this is the need for the application of low potentials, *i.e.* −3.05 to −1.45 V *vs.* SHE (0 to 1.5 *vs.* Li/Li^+^), to reduce Me^*n*+^ to Me^0^ in PBAs. For example, in the case of one of the common cathode materials K_2_Fe^II^[Fe^II^(CN)_6_] during positive polarization (regarding OCV), there is a reversible potassium intercalation reaction (eqn (1)) with a practical capacity of 90 mA h g^−1^ at 0.1 A g^−1^ (∼1C) and good cyclability up to 1000 cycles (voltage window: 2.4–4.4 V *vs.* Li/Li^+^).^7^

1 K_2_Fe^II^[Fe^II^(CN)_6_] − K^+^ − e^−^ ⇌ KFe^III^[Fe^II^(CN)_6_] − K^+^ − e^−^ ⇌ Fe^III^[Fe^III^(CN)_6_]

However, during negative polarization (*versus* OCV), there is a reversible conversion reaction forming metallic Fe (eqn (2)) that provides almost 400 mA h g^−1^ of initial capacity at 1C in a voltage range of 0 to 1.5 V (*vs.* Li/Li^+^) with the following slow degradation during 100 cycles (capacity retention: ∼50%).^8^

2 KFe^II^Fe^III^(CN)_6_ + 5Li^+^ + 5e^−^ ⇌ 2Fe^0^ + KCN + 5LiCN

The demonstrated eqn (2) (ref. 8) is very simplified and in fact consists of several consecutive steps that should be investigated for a better understanding of the reaction mechanism.

One of the disadvantages of conversion reactions is the low reversibility of the 1^st^ reduction process due to the full structure and morphology changes, and the formation of a surface electrolyte interface (SEI) leads to poor coulombic efficiency of around 50%.^6,8,9^ Another drawback is the low cycling life that is usually limited to 100–200 cycles with the capacity fading down to 50–70% compared to the 2^nd^ cycle, which can be explained by the constant morphology changes followed by the loss of contact between the electrode and the current collector. Nevertheless, by applying a rational PBA representative, it is possible to achieve long cyclability at a high current density. It has been shown that K_*x*_Mn[Fe(CN)_6_] has a multistep mechanism of conversion involving the fracture and recombination of Mn (N-coordinated) bonds, while the stronger Fe (C-coordinated) bond is still preserved (eqn (3)–(7)).^6^

3 K_*x*_Mn^III/II^[Fe^III^(CN)_6_] + *x*Li^+^ + *x*e^−^ ⇌ Mn + Li_5_Fe^I^(CN)_6_ + KCN + LiCN

4 Mn + Li_5_Fe^I^(CN)_6_ − 2e^−^ − 2Li^+^ ⇌ Li_3_Mn^I^[Fe^II^(CN)_6_]

5 Li_3_Mn^I^[Fe^II^(CN)_6_] − e^−^ − Li^+^ ⇌ Li_2_Mn^II^[Fe^II^(CN)_6_]

6 Li_2_Mn^II^[Fe^II^(CN)_6_] − e^−^ − Li^+^ ⇌ LiMn^II^[Fe^III^(CN)_6_]

7 LiMn^II^[Fe^III^(CN)_6_] − e^−^ − Li^+^ ⇌ Mn^III^[Fe^III^(CN)_6_]

The K_*x*_Mn[Fe(CN)_6_]-based anode exhibits a reversible capacity of 480 mA h g^−1^ at a high current density of 1 A g^−1^ (∼2C) with a considerable cycling stability exceeding 1000 cycles, demonstrating that the right choice of transition metals in PBAs can significantly affect the reaction mechanism and its reversibility.

There are many examples of using conversion reactions in batteries with water-based electrolytes. Among them, silver–zinc and lead–acid batteries are the most famous and commercialized.^10–14^ Still, there is no electrode with a conversion type reaction based on PBA in water-based electrolytes due to the abovementioned reason of using low potentials (thermodynamic water instability). In this work, we study the possible application of the derivatives of the Prussian blue representative Ag_4_[Fe(CN)_6_].^15^ Ag_4_[Fe(CN)_6_] could potentially be used as an electrode material with the following reversible reaction (eqn (8)) (*E*^0^ = 0.1478 V *vs.* SHE).

8 Ag_4_[Fe(CN)_6_]_(solid)_ + 4e^−^ ⇌ 4 Ag_(solid)_ + [Fe(CN)_6_]_(soluble)_^4−^

However, the structural instability of Ag_4_[Fe(CN)_6_] in the dry state determined in this paper prevents its possible application. Nevertheless, here we show that derivatives obtained after Ag_4_[Fe(CN)_6_] decomposition with the inherent silver reduction/oxidation reaction have good reversibility in water-based electrolytes. The effect of certain cations and anions on the reaction reversibility of the synthesized material is investigated in detail.

## 2. Experimental

### 2.1 Material preparation

Ag_4_[Fe(CN)_6_] was synthesized by the recommended methodology.^16,17^ Briefly, 1.5 μmol K_4_[Fe(CN)_6_]·3H_2_O (Sigma Aldrich, ≥98.5%) and 6 μmol AgNO_3_ (Sigma Aldrich, ≥99.0%) were dissolved in 50 ml distilled water (separately), and the solution of AgNO_3_ was then added dropwise to the K_4_[Fe(CN)_6_] solution and purged by N_2_ to avoid iron oxidation. After full mixing of all reagents, a white precipitate was formed and aged for 30 min under stirring and N_2_ bubbling. The synthesized white product was washed with distilled water by multiple decantations (centrifugation, 5 rounds, 5000 rpm) and dried at 70 °C overnight under vacuum. The product exhibited a darkish-blue colour when fully dried.

### 2.2 Characterizations

The XRD data of the obtained product were collected using a D8 ADVANCE diffractometer (Bruker) (reflection geometry; Cu X-ray tube (*λ*_CuKα1_ = 1.54056 Å, *λ*_CuKα2_ = 1.54439 Å), Ni-filter; scintillation detector).

Morphological characterization was conducted using a Magellan XHR 400L field emission scanning electron microscope (FE-SEM) (FEI Company) equipped with an energy dispersive X-ray spectroscopy (EDS) detector (Oxford Instruments).

### 2.3 Electrochemical measurements

To evaluate the electrochemical behaviour of the synthesized material, a 3-electrode T-cell with glassy carbon current collectors were assembled (working electrode: material under investigation; reference electrode: Ag/AgCl; counter electrode: carbon cloth (Kynol Europa GmbH) and Celgard 5550 as a membrane). Electrodes were prepared by thorough mixing of the active material (80 wt%), carbon black as a conductive additive (10 wt%), and 5% polyvinylidene fluoride solution in N-methylpyrrolidone (10 wt%) in an agate mortar. The prepared slurry was casted on thin graphitic foil with drying at 100 °C overnight under vacuum. The average loading of the active materials was 1.1 mg cm^−2^. The measurements were performed using a potentiostat/galvanostat BioLogic VSP-300. Solutions of 1 M NaClO_4_ and 1 M LiCl were used as electrolytes. Before the cell assembly, vacuum impregnation of the working electrodes and the membrane by electrolyte was done in vacuum for 5 min.

## 3. Results and discussion

**Fig. 1:**
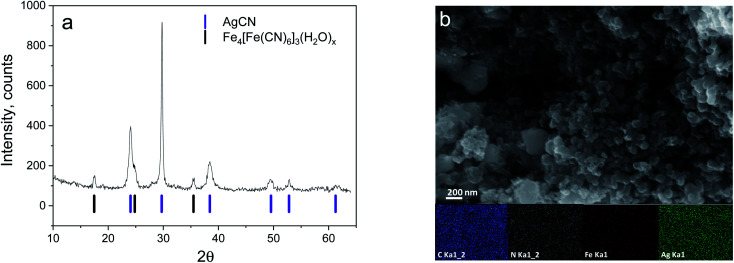
XRD pattern (a) and SEM image (b) of the synthesized material.

Usually, Ag_4_[Fe(CN)_6_] is prepared by precipitation, has a white colour, and is used in a wet state for sensing reaction, catalysis, and silver reduction.^16–18^ However, after moderate drying, the sample turned a darkish-blue, suggesting a change in its structure or even its composition. The structural composition of the obtained material was assessed by XRD analysis (Fig. 1a). The diffraction pattern shows the presence of two cubic phases: the first is AgCN and the second is likely to be a type of PBA, namely Fe_4_[Fe(CN)_6_]_3_(H_2_O)_*x*_.^19^ Apparently, the target material Ag_4_[Fe(CN)_6_] decomposes during the drying procedure leading to its separation into AgCN units and the formation of a new phase and a residual Fe_4_[Fe(CN)_6_]_3_(H_2_O)_*x*_ (dark-blue colour) phase, where the Fe cation comes from a partially torn apart [Fe(CN)_6_]^4−^ anion.

The fact that there is no structural information on Ag_4_[Fe(CN)_6_] in the literature (though the compound is mentioned^15^), neither in the crystallohydrate nor water-free form, supports the hypothesised very low stability of the target compound. Apparently, Ag_4_[Fe(CN)_6_] is unstable at elevated temperatures (70 °C) or in a semi-dry atmosphere and loses structural water very easily. Considering that in most cases, Me_4_[Fe(CN)_6_]-type structures simply do not have enough space to accommodate four cations per single hexacyanoferrate anion in the PBA framework, stabilization can be achieved only by the introduction of water as an additional structural element of the framework, resulting in the general formula M_4_[Fe(CN)_6_](H_2_O)_*x*_ (examples: K_4_[Fe(CN)_6_]·3H_2_O,^20^ Na_4_[Fe(CN)_6_]·10H_2_O (ref. 21)). Obviously, Ag_4_[Fe(CN)_6_](H_2_O)_*x*_ does not have enough stability to hold the structural water and simply decomposes into AgCN.

From a practical point of view, it is still interesting to investigate the electrochemical properties of the synthesized material, such as the reaction reversibility, practical capacity, and stability, due to the probable existence of two electroactive phases: the conversion-type reaction of silver reduction/oxidation from AgCN (*E*^0^ = −0.017 V *vs.* SHE) (eqn (9)) and the cation intercalation in the remaining PBA derivative.

9 AgCN + e^−^ ⇌ Ag + CN^−^

SEM images of the synthesized material show the presence of nano-sized (50–100 nm) particles agglomerated into micron-sized boulders (Fig. 1 and S1†). The observed morphology of the complex material is not uniform without separation into 2 phases. EDX mapping of the material also confirms the presence of Fe, C, N, and Ag without indication of K, indicating a good washing process and having a good agreement with the XRD-detected components (Fig. S1†).

Electrochemical testing of the synthesized material was performed in LiCl and NaClO_4_ 1 M solutions to check the reversible activity of the material for possible application in Li and Na aqueous batteries with different anions. The potential window was set from −0.75 to +0.75 V (*vs.* Ag/AgCl) to investigate the expected electrochemical reactions.

**Fig. 2:**
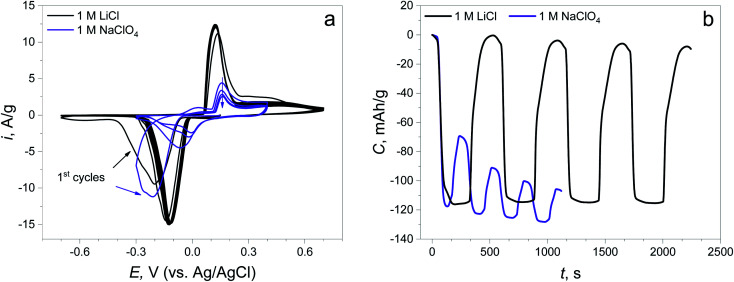
(a) CV curves of the synthesized material in 1 M LiCl and 1 M NaClO_4_ electrolytes at 5 mV s^−1^; (b) time capacity dependence curves obtained by CV curve integration.

The CV curves show that the reduction peaks occurring at the first negative polarization are almost at the same potential (−0.214 V) in both electrolytes, revealing the same nature of the first electrochemical reaction (Fig. 2a). The peaks of the reverse process (oxidation) are found at different potentials: 0.121 V in LiCl and 0.157 V in NaClO_4_, suggesting the changing of the route of electrochemical reaction. The potentials of the subsequent reduction cycles in both electrolytes are different for each of the electrolytes and are shifted to a more positive value compared to the initial point. This potential shift can signify the decreasing overpotential of the same reaction due to the change in material resistance and the changing of the reaction mechanism, which will be discussed later. Furthermore, there is almost no capacity degradation in the LiCl electrolyte during 5 cycles, while there is a fast capacity fading with low reversibility at the 1^st^ cycle in NaClO_4_ (capacity retention of the 1^st^ cycle: 99.8% in the LiCl electrolyte, 40.9% in the NaClO_4_ electrolyte, Fig. 2b).

**Fig. 3:**
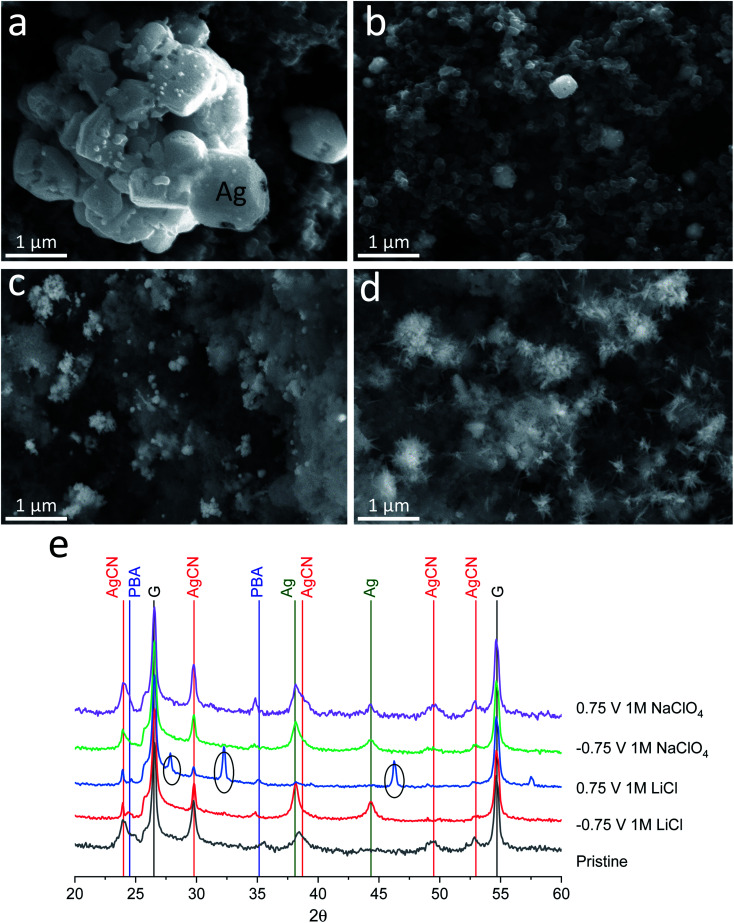
Post-mortem analysis of the charged and discharged electrodes in 1 M LiCl and 1 M NaClO_4_ electrolytes. SEM images of (a) electrodes charged up to −0.75 V in 1 M LiCl, (b) discharged up to 0.75 V in 1 M LiCl, (c) charged up to −0.75 V in 1 M NaClO_4_, and (d) discharged up to 0.75 V in 1 M NaClO_4_; (e) XRD patterns (PBA: Fe_4_[Fe(CN)_6_]_3_(H_2_O)_*x*_, G: graphitic foil current collector, peaks in circles: AgCl, intensity is taken as log(counts)).

To investigate the different behaviour of the same material in two different electrolytes and understand the effect of electrolyte components (cations and anions) on capacity fading, post-mortem analysis of the electrodes after the 1^st^ reduction (half cycle, charged) and after the reduction and oxidation processes (full cycle, discharged) was conducted by CV at 5 mV s^−1^ (Fig. S2†), followed by XRD and SEM analysis. The XRD and SEM experiments of the cycled electrodes were performed in a standard *ex situ* mode with the current collectors that affected the collected XRD data (additional intensive peaks of graphite foil, Fig. 3e).

SEM images of the electrodes after the electrochemical experiments in both electrolytes show significant differences in morphology and composition (Fig. 3a–d and S3†). After the first negative polarization to −0.75 V, a similar silver plating is observed on the electrodes. Both electrolytes (Fig. 3a and c) exhibit bright particles compared to the as-synthesized material (Fig. 1b). The process of silver plating occurs according to eqn (9) and should not be affected significantly by electrolyte cations and anions. However, major differences are visible after the reverse (oxidation) process to +0.75 V, wherein metallic silver should dissolve. In the case of the LiCl electrolyte, the morphology of the material fully returns to its initial state (Fig. 3b), while in the case of the NaClO_4_ electrolyte, dendrite-like particles form on the locations where metallic silver was plated (Fig. 3d).

To study the stability of the structures of the compounds on the electrode surface after the 1^st^ reduction cycle, XRD analysis of the electrodes was performed. It showed that the formation of metallic silver occurs in both electrolytes similarly after charging up to −0.75 V (Fig. 3e, green lines). When discharged up to 0.75 V, the electrode material in the 1 M NaClO_4_ electrolyte system demonstrates no significant difference in the diffraction patterns between the 0.75 V and −0.75 V states, suggesting very little structural changes happening over the charge/discharge process. However, a closer examination of the diffraction pattern hints at the relative increase of AgCN peaks, suggesting the formation of additional amounts of this phase (Fig. 3e, green and violet lines). Considering the formation of dendrite-like particles seen in the SEM images, we can surely say that this is AgCN. New electrochemically formed AgCN starts to grow from metallic silver particles, and due to lack of CN^−^ anions, (only released from the 1^st^ charge, eqn (9)), dendrite-like particles are formed. Since metallic Ag is still present in the material, we may conclude the incompleteness of the Ag oxidation process. In the case of the material treated in the 1 M LiCl electrolyte system in the same conditions (fully discharged), we observe the vanishing of the Ag phase and the presence of the AgCl phase (Fig. 3e, black circles). Since the CV curves for the 1 M LiCl cell have significant stability over multiple cycles, we may conclude that the process is highly reversible. Despite the fact that the solubility constant is higher for AgCl (*K*_sp_ = 1.8 × 10^−10^) than for AgCN (*K*_sp_ = 2.5 × 10^−16^), the high concentration of chloride ions apparently promotes the formation of AgCl, which can be further cycled reversibly according to the following equation:

10 AgCl + e^−^ ⇌ Ag + Cl^−^

It should be noted that Fe_4_[Fe(CN)_6_]_3_(H_2_O)_*x*_ does not undergo noticeable changes in the samples during the electrochemical reaction and dissolution in the electrolytes (Fig. 3e, blue line, PBA).

These findings show that the capacity degradation of the AgCN material in perchlorate electrolyte is conditioned by the lack of CN^−^ diffusing to the electrolyte bulk (eqn (9)). Less than 41 wt% of the material is able to transform into the initial form after the 1^st^ cycle following fast degradation. Meanwhile, there is negligible capacity fading in the chloride electrolyte due to the formation of AgCl and its known reversible reaction (eqn (10)).^22,23^

**Fig. 4:**
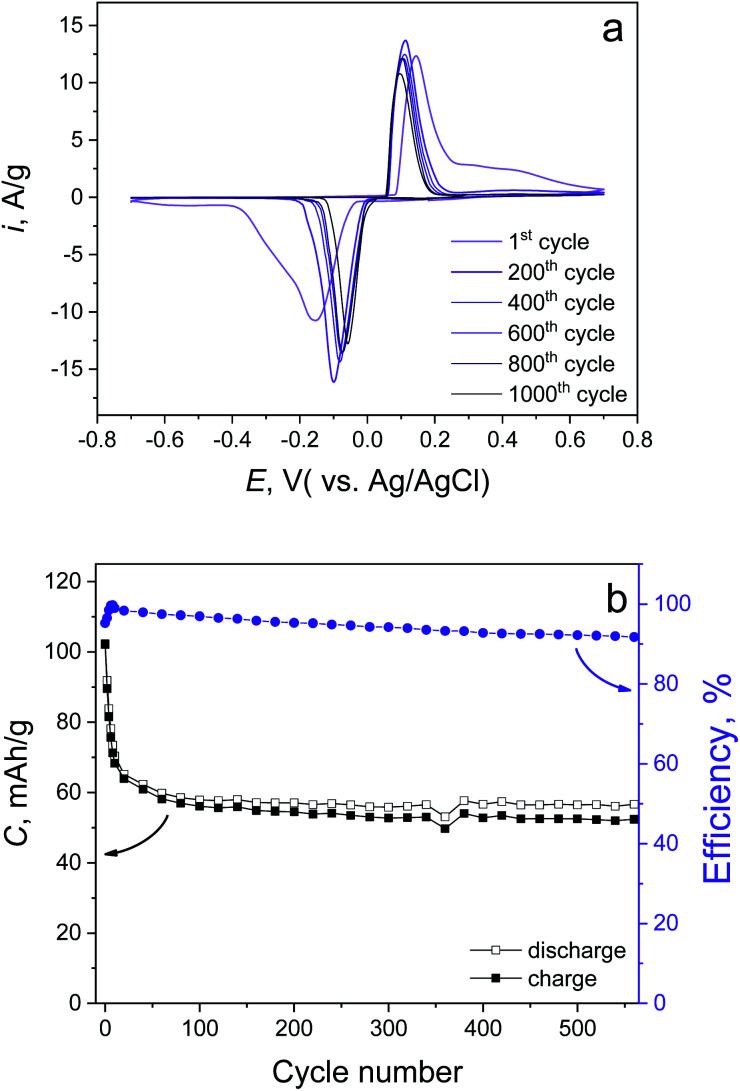
Cycling stability tests of the synthesized material performed by (a) CV mode at 5 mV s^−1^ and (b) GC mode at 1 A g^−1^.

In order to check the cycling life and rate capabilities, the synthesized material was further investigated in 1 M LiCl electrolyte. The CV curves obtained at various scan rates (Fig. S4a†) demonstrate the high-rate capability of the conversion reaction (eqn (10)). Even at 50 mV s^−1^, there is 70% remaining capacity compared with the 1 mV s^−1^ case (79.7 and 114 mA h g^−1^, respectively, Fig. S4b†). Cycling stability tests of the synthesized material have been done by CV measurement (Fig. 4a) at 5 mV s^−1^, which revealed a 50.1% capacity fading up to 1000 cycles, which is fully confirmed by prolonged galvanostatic cycling (GC) at 1 A g^−1^ (10C, positive and negative voltage cut offs are +0.75 and −0.4 V, respectively). This also demonstrates 50.5% capacity degradation up to 500 cycles (Fig. 4b). We believe that the stability of the prolonged cycling conditioned only by Cl^−^ ions does not depend on the cation (eqn (10)). This suggests the use of different soluble chlorides (Li^+^, Na^+^, K^+^).

## 4. Conclusions

We have shown the instability of the Ag_4_Fe(CN)_6_ material, which can be possibly used as an electrode material with a conversion reaction mechanism. Me_4_[Fe(CN)_6_]-type structures are usually stabilized by the introduction of water molecules as an additional structural element for the possibility of the accommodation of four cations per hexacyanoferrate anion in the PBA framework. At elevated temperatures, Ag_4_[Fe(CN)_6_](H_2_O)_*x*_ does not have enough stability to hold structural water and it decomposes into AgCN and a residual iron cyanide complex. It was shown that the decomposition products of Ag_4_[Fe(CN)_6_] have electrochemical activity due to the reversible oxidation/reduction of Ag compounds in aqueous electrolytes. Electrodes based on the synthesized material show good reversibility in Cl^−^-containing electrolytes due to the formation of AgCl. The synthesized material based on AgCN (derivative of Ag_4_Fe(CN)_6_) can be used as a stable counter electrode in Cl^−^-containing electrolytes for battery material investigation. We believe that it is possible to find an appropriate PBA representative (with a suitable transition metal that can be reduced at low potentials) with a reversible conversion reaction mechanism for aqueous-based batteries that can provide more power and energy density compared to conventional PBAs with the intercalation mechanism.

## Author contributions

Fyodor Malchik: conceptualization, methodology, supervision. Netanel Shpigel: conceptualization, methodology. Kaiyrgali Maldybayev: formal analysis. Tatyana Kan: formal analysis. Saule Kokhmetova: formal analysis. Andrey Kurbatov: supervision. Alina Galeyeva supervision. Nufar Tubul: formal analysis. Thierry Djenizian: conceptualization, methodology.

## Conflicts of interest

There are no conflicts to declare.

## Supplementary Material

RA-012-D2RA00617K-s001
