# Supplementary material for: Application of a conversion electrode based on decomposition derivatives of Ag4[Fe(CN)6] for aqueous electrolyte batteries
Source: RSC Adv. 2022 Mar 29;12(16):9862–7. doi: 10.1039/d2ra00617k (PMC8963386; doi:10.1039/d2ra00617k)
Supplement: RA-012-D2RA00617K-s001 [file RA-012-D2RA00617K-s001.pdf]

This journal is © The Royal Society of Chemistry 2022

Supplementary material

Application of conversion electrode based on decomposition derivatives of $Ag_4[Fe(CN)_6]$ for aqueous electrolyte batteries

Fyodor Malchik,*[a] Kaiyrgali Maldybayev,[a] Tatyana Kan,[a] Saule Kokhmetova,[a] Andrey Kurbatov,[a] Alina Galeyeva,[a] Nufar Tubul,[b] Netanel Shpigel [c] and Thierry Djenizian [a,d]

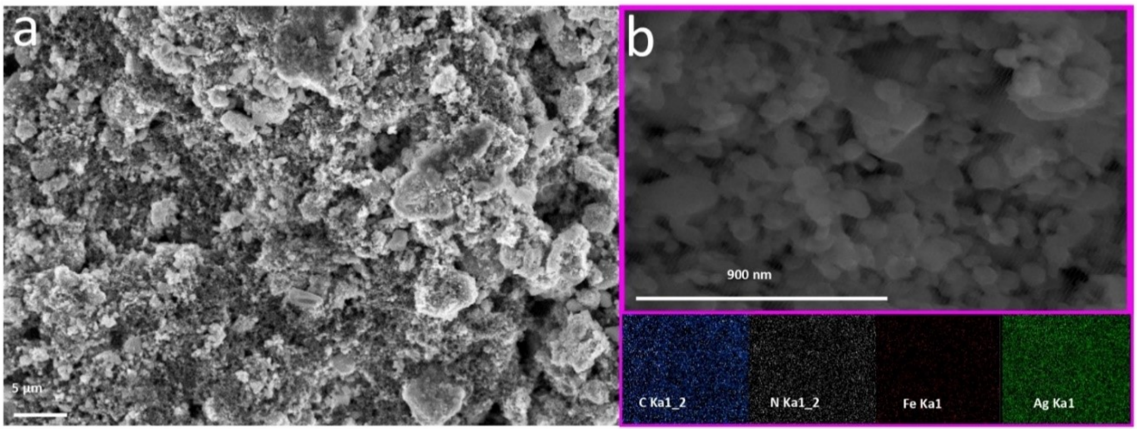

Fig. S1 SEM images of synthesized material at different magnification and EDX mapping conducted with the image at low magnification

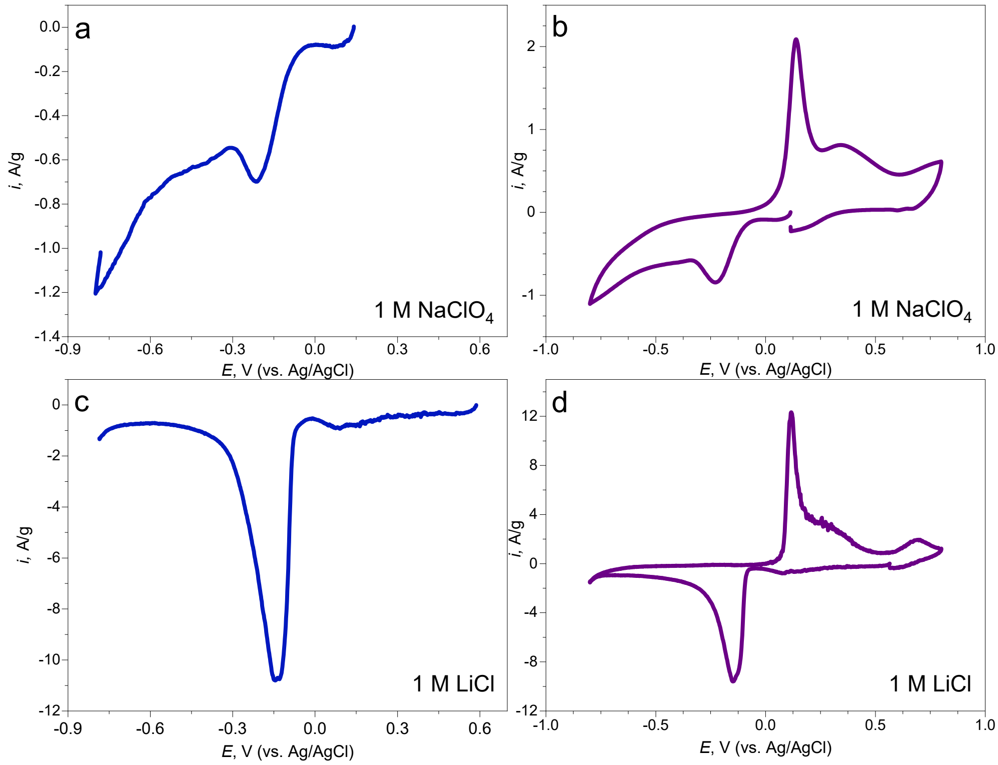

a. al-Farabi Kazakh National University, Center of Physical-Chemical Methods of Research and Analysis, Almaty, 050012, Kazakhstan.
b. Department of Chemistry and BINA – BIU Center for Nanotechnology and Advanced Materials, Bar-Ilan University, Ramat-Gan 5290002, Israel.
c. Institute of Chemistry, The Hebrew University of Jerusalem, Jerusalem 9190401, Israel.
d. Mines Saint-Etienne, Center of Microelectronics in Provence, Flexible Electronics Department, 13541, Gardanne, France.
* corresponding author.
Electronic Supplementary Information (ESI) available: [details of any supplementary information available should be included here]. See DOI: 10.1039/x0xx00000x

Fig. S2 CV curves of synthesized material for postmortem analysis cycling from OCV at 5 mV/s: a) to -0.75 V (half cycle) in 1M $NaClO_4$; b) to -0.75 V and back to +0.75 V (full cycle) in 1M $NaClO_4$; c) to -0.75 V (half cycle) in 1M LiCl; d) to -0.75 V and back to +0.75 V (full cycle) in 1M LiCl

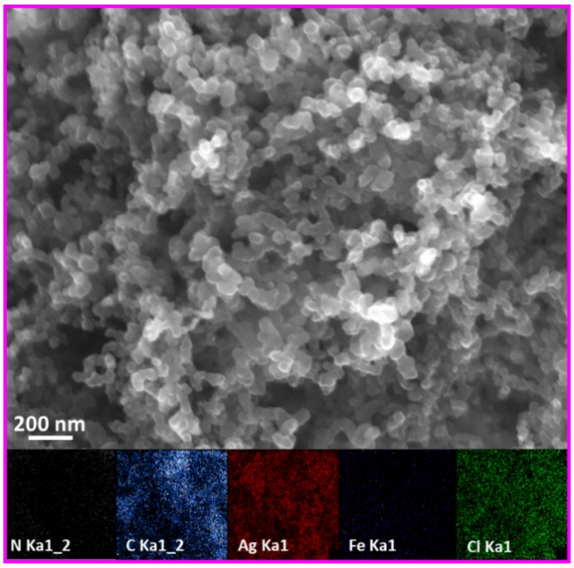

Fig. S3 SEM images of electrode after full CV cycle in 1M LiCl electrolyte and EDX mapping

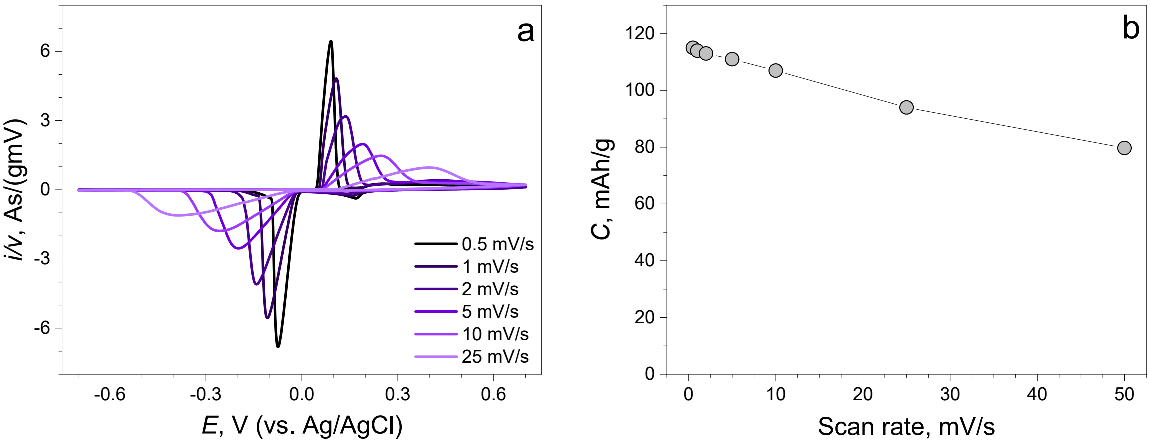

Fig. S4 a) CV curves of synthesized material in 1M LiCl electrolyte at different scan rates; b) capacity scan rate dependence curve.
